# Comparative Analysis of the Occurrence and Role of CX3CL1 (Fractalkine) and Its Receptor CX3CR1 in Hemophilic Arthropathy and Osteoarthritis

**DOI:** 10.1155/2020/2932696

**Published:** 2020-08-20

**Authors:** Piotr Wojdasiewicz, Łukasz A. Poniatowski, Andrzej Kotela, Marta Skoda, Michał Pyzlak, Aleksandra Stangret, Ireneusz Kotela, Dariusz Szukiewicz

**Affiliations:** ^1^Department of General and Experimental Pathology, Centre for Preclinical Research and Technology (CePT), Medical University of Warsaw, Pawińskiego 3C, 02-106 Warsaw, Poland; ^2^Department of Rehabilitation, Eleonora Reicher National Institute of Geriatrics, Rheumatology and Rehabilitation, Spartańska 1, 02-637 Warsaw, Poland; ^3^Department of Experimental and Clinical Pharmacology, Centre for Preclinical Research and Technology (CePT), Medical University of Warsaw, Banacha 1B, 02-097 Warsaw, Poland; ^4^Department of Neurosurgery, Maria Skłodowska-Curie Memorial Cancer Center and Institute of Oncology, W. K. Roentgena 5, 02-781 Warsaw, Poland; ^5^Department of Orthopedics and Traumatology, 1st Faculty of Medicine, Medical University of Warsaw, Lindleya 4, 02-005 Warsaw, Poland; ^6^Department of Orthopedics and Traumatology, Central Clinical Hospital of the Ministry of the Interior and Administration, Wołoska 137, 02-507 Warsaw, Poland; ^7^Department of Rehabilitation in Disease of the Locomotor System, Faculty of Medicine and Health Sciences, Jan Kochanowski University, Kielce, Poland

## Abstract

**Objective:**

Hemophilic arthropathy is characterized by recurrent bleeding episodes in patients with hemophilia leading to irreversible joint degeneration. The involvement of CX3CL1 (fractalkine) and its receptor CX3CR1 was observed in the pathogenesis of numerous arthritis-associated diseases. Taking this into account, we have presented a study investigating the role of the CX3CL1/CX3XR1 axis in the course of hemophilic arthropathy, including the CX3CL1-dependent expression of CD56^+^, CD68^+^, and CD31^+^ cells along with evaluation of articular cartilage and synovial membrane morphology.

**Methods:**

The study was carried out using cases (*n* = 20) of end-stage hemophilic arthropathy with a severe type of hemophilia A and control cases (*n* = 20) diagnosed with osteoarthritis. The biofluids including blood serum and synovial fluid were obtained intraoperatively for the evaluation of CX3CL1 using the ELISA test. Tissue specimens including articular cartilage and synovial membrane were similarly collected during surgery and stained immunohistologically using selected antibodies including anti-CX3CR1, anti-CD56, anti-CD68, and anti-CD31. Additionally, the analysis included the assessment of articular cartilage, synovial membrane, and blood vessel morphology.

**Results:**

In our study, we have documented increased average concentration of CX3CL1 in the blood serum of the study group (7.16 ± 0.53 ng/ml) compared to the control group (5.85 ± 0.70 ng/ml) without statistically significant difference in synovial fluid concentration at the same time. We have observed an increased macrophage presence with more marked proliferation and fibrosis of the synovial membrane in the study group. Remaining results such as expression of CX3CR1 presence of NK cells and larger surface area of blood vessels within the synovial membrane were noted also without statistical significance.

**Conclusions:**

This study has demonstrated collective CX3CL1/CX3CR1 axis involvement in hemophilic arthropathy pathogenesis introducing new interesting diagnostics and a therapeutic target.

## 1. Introduction

The pathophysiology of hemophilia covers a sex-linked inherited coagulation disorder caused by the reduced levels of plasma glycoproteins including the coagulation factor VIII (FVIII) leading to the development of hemophilia A and the analogous reduced levels of coagulation factor IX (FIX) leading to the development of hemophilia B (Christmas disease) [1]. Hemarthrosis consists one of the most common complications in the course of congenital coagulation disorders [2]. It is estimated that they account for 70-80% of all hemorrhages in patients with hemophilia [3]. Recurrent clinical and subclinical hemorrhages into the musculoskeletal system lead to the irreversible changes revealing as pain, rigidity, and marked limitation of joint mobility [2, 3]. The syndrome of articular manifestations in the course of hemophilia is known as hemophilic arthropathy (HA) [2]. According to the literature, ~90% of patients with a severe type (FVIII < 0.01 IU/ml in serum) of hemophilia A experience hemorrhage with at least one joint bleeding at the age of 4.4 years [4]. The effective prophylactic factor replacement therapy (FRT) is available in many regions of the world, including highly developed countries [3]. However, advanced and fully developed HA still occurs worldwide. In the most severe cases of congenital bleeding disorders, even regular administration of FRT may not prevent the progression of the disease due to the development of plasma inhibitors (anti-FVIII/FIX) against epitopes of appropriate clotting factors [5]. Therefore, addressing the problems of the pathogenesis, prevention, and modern strategies of HA treatment appear to be still justified. There are numerous studies available in the professional literature regarding to the pathophysiology of HA. However, it has not been fully elucidated, especially as regards the pathomechanisms which are associated with the early stage of the disease and, to be more precise, with subclinical early and chronic joint inflammation. The direct presence of extravasated blood in the joint triggers an inflammation which initiates subsequent immunopathological mechanisms [6]. The extravasated blood contains numerous morphotic elements which remain active as regards the secretion of inflammatory mediators including the granulocytes, monocytes, and lymphocytes [7]. Over time, the blood and its related metabolites are progressively absorbed by macrophages (M*Φ*) and synovial membrane cells [2]. The ability of blood metabolite removal by immune system cells and the synovial membrane during recurrent episodes of hemarthrosis decreases progressively [2]. In consequence, the prolonged presence of toxic blood metabolites occurs, such as iron (Fe^2+^) and deposition of hemosiderin in the synovial membrane cells [8]. This, in turn, leads to the number of pathophysiological events including the articular cartilage and subchondral bone degeneration, fibroblast proliferation, and angiogenesis collectively followed by the synovial membrane hypertrophy [2]. Collectively, the mentioned data concerning pathophysiology of HA and other arthritis-related diseases indicate that the degeneration of joint tissues within the musculoskeletal system is interconnected with persisting and chronic inflammatory state [6]. Cytokines seem to play a leading, but not completely clarified, role among various compounds participating in the induction and circuit-specific inflammatory processes in the pathogenesis of HA [9]. This heterogeneous group of proteins has a number of features including the activation and proliferation of immune system cells resulting in functional influence of various types and lines of cells through multilevel and complex system of interconnections known as a cytokine network [10]. Furthermore, what is relevant in this context including the direct and indirect influences of cells which compose the joint and likely infiltrate its structures via vasculature and lymphatics [11]. Respecting the wide range of inflammatory mediators associated with the musculoskeletal system diseases, the increasing role and attention have been attributed to the chemokines in recent years due to their specific structural and chemoattractive properties [12]. Currently, almost 50 chemokine ligands have been identified, out of which CX3CL1 (fractalkine) and its receptor CX3CR1 deserve certain attention according to its unique features [13]. Except for its core chemotactic properties, CX3CL1 has a different molecular biology and could function as an adhesive molecule facilitating additional mechanism concerning accumulation of immune system cells at the site of ongoing inflammation [14, 15]. Within the pathologies of the musculoskeletal system, the role of the CX3CL1/CX3CR1 axis was to date associated with development of conditions that include, but are not limited to, osteoarthritis (OA), rheumatoid arthritis (OA), and degenerative disc disease (DDD) [16, 17]. However, to date, the role of the CX3CL1/CX3CR1 axis has not been widely discussed in the wider context of its presence and possible pathophysiological functions in the course of HA. The chronic nature of this pathology associated with the frequent hemarthroses followed by ongoing joint destruction potentially appears to be strongly associated with the pathomechanisms occurring with the participation of the CX3CL1/CX3CR1 axis [18, 19]. Reflecting on this, we hypothesize that the elucidation of the possible role of the CX3CL1/CX3CR1 axis in the pathogenesis of HA could provide a new potential supplementary diagnostic marker or therapeutic target [20]. According to these facts, we conducted a study in an attempt to evaluate CX3CL1 concentration levels in the serum and synovial fluid along with expression of CX3CR1 in the articular cartilage and synovial membrane in patients with HA and OA which served as a comparative control group. We extended our study by performing immunohistochemical and histological stainings within articular cartilage and synovial membrane for assessment of the CX3CL1-dependent expression of CD56 (Cluster of Differentiation 56), CD68 (Cluster of Differentiation 68), and CD31 (Cluster of Differentiation 31) cell surface markers along with evaluation of articular cartilage and synovial membrane morphology. Based on the literature review, there is no similar study that has been previously published. The results of this study that pertain to the evaluation of a possible role of the CX3CL1/CX3CR1 axis in the pathogenesis of HA have been presented in this manuscript.

## 2. Materials and Methods

### 2.1. Study Design and Patient Population

Collectively, the study included a total of 40 patients (*n* = 40) which were divided into two consecutive groups enrolled over the two-year period between 2013 and 2015 which were divided into two consecutive groups. The qualification process was based on the medical history, clinical assessment, radiological evaluation, and other supplementary tests performed before elective total joint replacement surgery [21, 22]. All procedures were performed in accordance with the ethical standards of the responsible committee on human experimentation and with the Helsinki Declaration of 1975, as revised in 2000. The study protocols were approved by the Ethics Committee of the Central Clinical Hospital of the Ministry of the Interior in Warsaw (67/2013). The informed consent was consecutively obtained from all of the study participants. According to this, the study group consists of 20 male individuals (*n* = 20) with a severe type (FVIII < 0.01 IU/ml in serum) of hemophilia A diagnosed with end-stage HA, radiologically assessed as grade 4 due to the Kellgren-Lawrence scale. These included 13 patients (*n* = 13) qualified for primary total knee arthroplasty (TKA), 6 patients (*n* = 6) qualified for primary total hip arthroplasty (THA), and 1 patient (*n* = 1) qualified for primary total ankle arthroplasty (TAA). The estimated average age for the study group was 37.4 years (with the youngest case being 26 years and the oldest 53 years). The average period of time between qualification for the surgery and the procedure was 1.5 years (ranged from 0.5 to 3 years). Among the study group, 3 patients (*n* = 3) were known to be seropositive for hepatitis C virus (HCV). No presence of anti-FVIII was detected. The control group (*n* = 20) consists of 15 male and 5 female individuals diagnosed with end-stage OA, radiologically assessed in 19 patients (*n* = 19) as grade 4 and in 1 patient (*n* = 1) as grade 3 due to the Kellgren-Lawrence scale. These included 12 patients (*n* = 12) who qualified for primary TKA and 8 patients (*n* = 8) who qualified for primary THA. The estimated average age for the study group was 48.6 years (with the youngest case being 44 years and the oldest 61 years). The average period of time between the qualification for the surgery and the procedure was 3.5 years (ranged from 1.5 to 6 years). Among the control group, 1 patient (*n* = 1) was known to be seropositive for HCV.

### 2.2. Biofluid and Tissue Specimen Obtainment and Preparation

The procedure for each patient covers the collection of two types of material for examination. The first type consisted of biofluids including the serum and synovial fluid which were obtained for evaluation of the CX3CL1 occurrence and concentration. Directly prior to the orthopedic surgery procedure, in the operating room, venous blood was sampled from the area of the cubital fossa from the study and control group patients with a set including a disposable needle (4657527; Sterican 21G × 1 1/2 in Hypodermic Needle, B. Braun, Melsungen AG, Germany), syringe (4606108V; Injekt 10 ml Eccentric Syringe, B. Braun, Melsungen AG, Germany), and 2.7 ml sterile test tube (34ML-0275-4A; Medlab Products, Poland) consisting of 0.3 ml of a 2.3% solution of sodium citrate (C_6_H_5_Na_3_O_7_). Then, directly after preparing the operating field, the sterile procedure was performed to aspirate synovial fluid from the affected joint with a set including a disposable needle (4665120; Sterican 18G × 1 1/2 in Hypodermic Needle, B. Braun, Melsungen AG, Germany), syringe (4606108V; Injekt 10 ml Eccentric Syringe, B. Braun, Melsungen AG, Germany), and 4 ml test tube (34ML-0405-7A; Medlab Products, Poland) consisting of 50 *μ*l of dispersed lithium heparin (18 IU/ml). The venous blood was obtained from all of the patients from both the study and control groups. Therefore, synovial fluid was collected from 15 patients (*n* = 15) from the study group and 15 patients (*n* = 15) from the control group. The lower number of collected samples of synovial fluid compared to the number of serum samples resulted from the occurring lack of synovial fluid in the operated joints in both groups of patients. The test tubes with venous blood and synovial fluid were stored at 4°C until the orthopedic surgeons obtained the second type of material (no longer than 20 minutes since the surgery started) which consisted of articular cartilage and synovial membrane used for immunohistochemical and histological stainings. The obtained fragments of the articular cartilage and synovial membrane were secured in separate sterile 20 ml containers for histopathological tests (EM-100020; Elektro Med, Poland) with 10% aqueous (H_2_O) solution of formaldehyde (CH_2_O) added as a preservative. Subsequently, serum and synovial fluid were stored at -80°C until the end of the time of tests. The tissue specimens obtained intraoperatively were stored at -15°C before further proceeding.

### 2.3. Evaluation of the CX3CL1 Concentration in Biofluids

The quantitative occurrence and concentration of CX3CL1 were evaluated in obtained biofluid samples including the serum and synovial fluid using an enzyme-linked immunosorbent assay (ELISA) test. Followed assays and labeling were carried out using the Human Fractalkine (CX3CL1) ELISA kit (ab100522; Abcam, Boston, MA, USA), following the standard manufacturer protocol. Both the serum and synovial fluid were twice diluted with appropriate assay diluent provided with the kit. The synovial fluid was additionally incubated for 15 min at 37°C with the addition of hyaluronidase (H3506; Sigma, St. Louis, MO, USA) at the concentration of 10 mg/ml to improve the reliability of the results [23]. Consecutively, the samples were centrifuged at 800 rpm for 10 min with X-column test tubes (Costar 8169; Corning, Amsterdam, Netherlands). Subsequently, biofluid samples and standards of 100 *μ*l were incubated in an appropriate 96-well microplate precoated with specific second-generation antibody labeled with streptavidin- (SA-) horseradish peroxidase (HRP) conjugate. The photometric assessment concerning the absorbance of the resulting color product was measured within 2 hours at a wavelength of 450 nm using Asys UVM340 Microplate Reader (G019065090; Biochrom, Cambridge, UK). The interpretation of obtained CX3CL1 concentrations was determined using the standard curve comprising results obtained from the previously prepared standards attached to the kit. For all the samples, the minimum detectable concentration was 0 ng/ml, and all results below were assigned as 0 ng/ml. Each test sequence was assayed in the two serial repetitions, and consequently, the average results were used for the analysis.

### 2.4. Immunohistochemical and Histological Methods

The immunohistochemical and histological assessment included a total of 80 obtained tissue specimens. Preparation of the collected tissue material covered cutting to the size of 15 mm × 15 mm × 3 mm while articular cartilage fragments which included osseous tissue were first decalcified in EDTA (C_10_H_16_N_2_O_8_) solution for ~12 hours. Subsequently, tissue specimens were embedded in liquid paraffin in standard histology cassettes (C31050101Y; Elektro Med, Poland) with appropriate chemical reagents and placed in the automatic tissue processor (TP1020; Leica Microsystems, Nussloch, Germany). Therefore, material was gradually dehydrated by immersing in gradually concentrated ethanol (C_2_H_5_OH) solutions such as 70%, 95%, and 100%. Subsequently, the tissue specimens were immersed in xylene (C_8_H_10_) and embedded in paraffin (P3558; Sigma, St. Louis, MO, USA) following forming into paraffin blocks ready for microtome processing. The prepared blocks were cut with a semimotorized rotary microtome (RM2245; Leica Microsystems, Nussloch, Germany) for 4 *μ*m thickness slices. The specimen slices were applied on the standard slides (AA00000112E/J2800AMNZ; Thermo Scientific, Braunschweig, Germany) and then dried for ~1 hour at 37°C, deparaffinized, and rehydrated. Gradually, the obtained intact articular cartilage and synovial membrane specimens were stained histologically with hematoxylin and eosin (H&E). The immunohistochemical stains were performed with the selected antibodies including anti-CX3CR1 (ab184678; Abcam, Boston, MA, USA), anti-CD56 (IR628; Dako, Copenhagen, Denmark), anti-CD68 (GA609; Dako, Copenhagen, Denmark), and anti-CD31 (GA610; Dako, Copenhagen, Denmark). All the subsequent steps were performed using the EnVision FLEX High pH (K8000; Dako, Copenhagen, Denmark) system, and processed on an Autostainer Link 48 device (AS480; Dako, Copenhagen, Denmark). The preparations were sealed with cover slips (BBAD02400400#A1; Thermo Scientific, Braunschweig, Germany) and underwent proper assessment. The microphotographs of the tissue sections were taken using the Olympus BX53 microscope (Olympus Optical, Tokyo, Japan) equipped with the visual track and Olympus XC50 camera (Olympus Optical, Tokyo, Japan). The analysis was performed with Fiji/ImageJ software (version 1.49a; NIH, Bethesda, MD, USA) at standard magnifications including 40x, 100x, 200x, and 400x. The performed immunohistochemical stainings was used to evaluate consecutively the expression of CX3CR1 in the articular cartilage and synovial membrane (200x), evaluate the expression of CD56 antigens (NK cell-specific) and CD68 antigens (macrophage-specific) in the synovial membrane (400x), and evaluate the number and surface area of blood vessels via labeling of the CD31 antigen (vascular endothelium-specific) where quantification covers the average of three high power fields (HPF) rounded to integers (100x), and the surface area of vessels was calculated as a fraction of the total surface area in the synovial membrane. Furthermore, the analysis included the evaluation of articular cartilage degeneration and fibrosis (200x) along with the evaluation of the proliferation and fibrosis of synovial membrane (200x). An author-based semiquantitative scale was proposed and implemented for the assessment of the analyzed parameters per HPF (Table 1). Initially, the percentage (*X*) of the surface occupied by the changed tissue was assessed relative to the surface area of the entire preparation according to the following formula:

(1) $$\begin{array}{c} X=\frac{\text{Surface of changed cartilage}/\text{synovium per HPF}}{\text{Total surface of cartilage}/\text{synovium per HPF}}\times 100\% \end{array}$$

Then, cut-off points were set, which determined five grades of applied scale.

### 2.5. Statistical Analysis

The statistical processing of the obtained data was performed using Statistica 12.0 PL (StatSoft Inc., Tulsa, OK, USA) software and R statistical environment (version 3.2.5; R Development Core Team, Vienna, Austria). The detailed description of the applied methods and the underlying principles are described in the subsequent Results sections. The results were considered statistically significant when *p* values were less than adjusted 0.05 (*p* < 0.05).

## 3. Results

### 3.1. Concentration of the CX3CL1 in Biofluids

**Figure 1:**
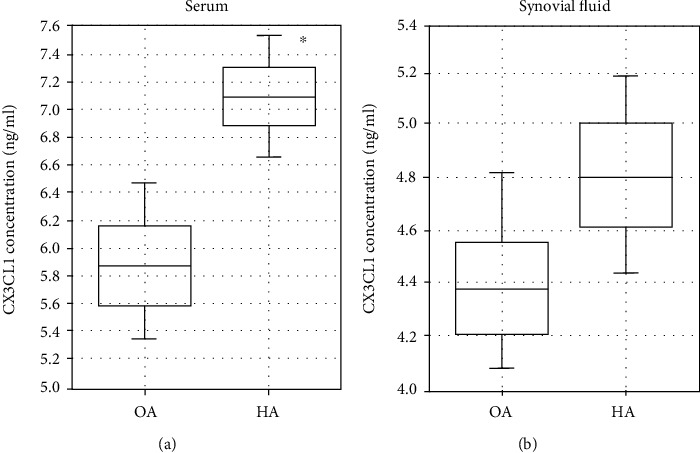
The average concentration of the CX3CL1 in serum (a) and synovial fluid (b) obtained from patients. The data has been presented as a mean value ± standard deviation (SD). ^∗^*p* < 0.05. OA: osteoarthritis; HA: hemophilic arthropathy.

According to the obtained data, the average concentration of CX3CL1 in the serum was elevated in the study group (7.16 ± 0.53 ng/ml) of HA patients compared to the control group (5.85 ± 0.70 ng/ml) of OA patients which was a statistically significant (*p* = 0.000078) difference (Figure 1(a)). At the same time, no statistically significant (*p* = 0.251) difference in synovial fluid concentration of CX3CL1 was observed in the study group (4.81 ± 1.39 ng/ml) of HA patients compared to the control group (4.38 ± 1.08 ng/ml) of OA patients (Figure 1(b)). Furthermore, according to the assumed hypothesis, the concentrations of CX3CL1 in the serum and synovial fluid have a compliant and normal distribution of assessed variables and were analyzed with the Student *t*-test.

### 3.2. Immunohistochemical and Histological Stainings

#### 3.2.1. Comparative Assessment of the CX3CR1 Expression

**Figure 2:**
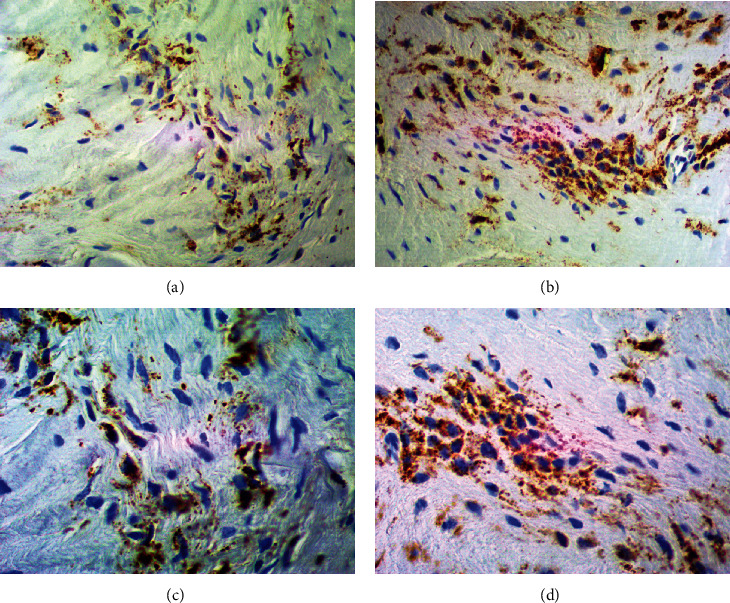
The representative microphotographs showing CX3CR1 expression in examined synovial membrane slices. (a) Synovial membrane from hemophilic arthropathy patient (×200 magnification). (b) Synovial membrane from osteoarthritis patient (×200 magnification). (c) Synovial membrane from hemophilic arthropathy patient (×400 magnification). (d) Synovial membrane from osteoarthritis patient (×400 magnification).

**Figure 3:**
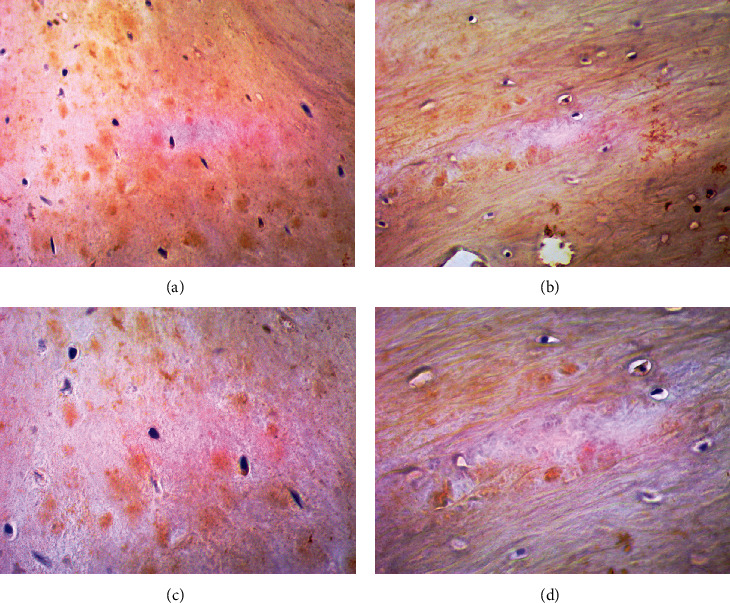
The representative microphotographs showing CX3CR1 expression in examined articular cartilage slices. (a) Articular cartilage from hemophilic arthropathy patient (×200 magnification). (b) Articular cartilage from osteoarthritis patient (×200 magnification). (c) Articular cartilage from hemophilic arthropathy patient (×400 magnification). (d) Articular cartilage osteoarthritis patient (×400 magnification).

The expression of CX3CR1 was positively observed in both analyzed groups within two types of the obtained tissues. In the synovial membrane was revealed the distinctly increased CX3CR1 expression in both the study and control groups which indicates its overexpression in this type of obtained material, especially towards the synoviocytes and fibroblasts (Figure 2). Furthermore, the significant changes and overexpression of CX3CR1 in articular cartilage was not observed between the study and control groups (Figure 3). We observed the slight tendency towards excessive CX3CR1 staining intensity of the extracellular matrix without significant intergroup differences.

#### 3.2.2. Comparative Assessment of the CD56 and CD68 Antigen Expression

**Figure 4:**
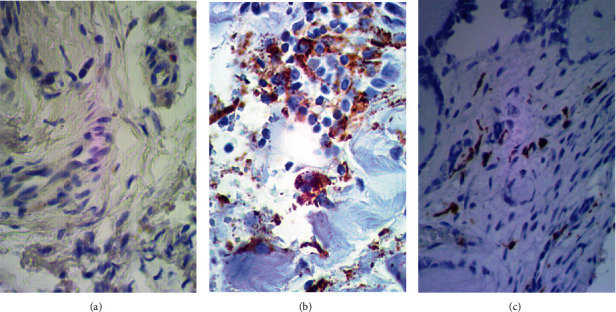
The representative microphotographs showing CD56 and CD68 antigen expression in examined synovial membrane slices. (a) Synovial membrane from hemophilic arthropathy patient (×400 magnification) immunostained with anti-CD56 antibody. (b) Synovial membrane from hemophilic arthropathy patient (×400 magnification) immunostained with anti-CD68 antibody. (c) Synovial membrane from osteoarthritis patient (×400 magnification) immunostained with anti-CD68 antibody.

**Figure 5:**
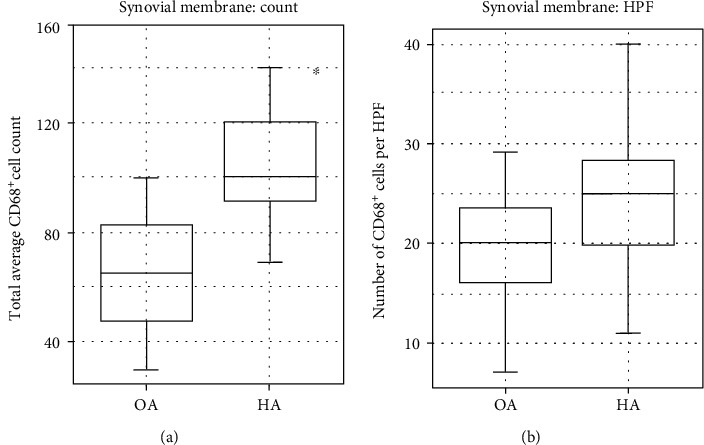
The average count of the CD68^+^ cells (a) and its number per HPF (b) in the synovial membrane. The data has been presented as a mean value ± standard deviation (SD). ^∗^*p* < 0.05. OA: osteoarthritis; HA: hemophilic arthropathy.

The analysis of the presence of immune system cells in the articular cartilage and synovial membrane was performed with specific antibodies against surface markers of macrophages (CD68^+^) and NK cells (CD56^+^) population (Figure 4). Both the articular cartilage and synovial membrane obtained from the control group patients showed no presence of NK cells. Analogous immunoreactivity of the CD56 antigen was observed in the articular cartilage obtained from the study group patients. Solitary CD56^+^ cells were noted only in the synovial membrane in the study group patients as 0.8 of the cell per HPF. According to the immunoreactivity of the CD68 antigen, both the total macrophage and the cell per HPF count were higher in study group patients (Figure 5). The average total count of CD68^+^ cells was estimated at 102.5 ± 21.18 and as 25.0 ± 7.04 of the cell per HPF in the synovial membrane obtained from study group patients. Consecutively, the average total count of CD68^+^ cells was estimated at 68.5 ± 29.25 and as 20.3 ± 7.97 of the cell per HPF in the synovial membrane obtained from control group patients. Welch *t*-test was conducted in order to compare the number of CD68^+^ cells in the synovial membrane of two investigated groups. Statistical analysis showed statistically significant (*p* < 0.001) difference only for the total number of CD68^+^ cells.

#### 3.2.3. Comparative Assessment of the Number and Surface Area of Blood Vessels

**Figure 6:**
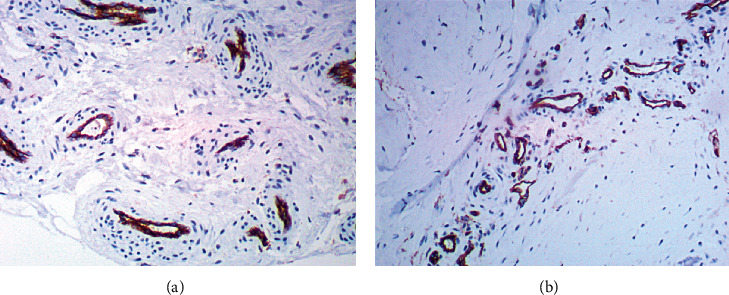
The representative microphotographs showing CD31 antigen expression in examined synovial membrane slices. (a) Synovial membrane from hemophilic arthropathy patient (×100 magnification). (b) Synovial membrane from osteoarthritis patient (×100 magnification).

**Figure 7:**
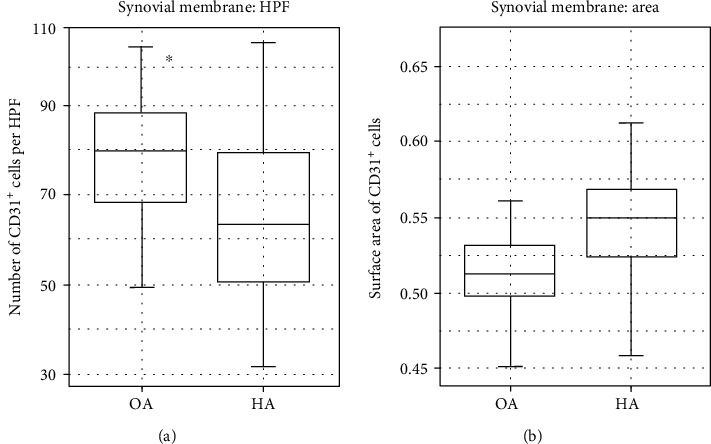
The average number of the CD31^+^ cells per HPF (a) and its occupied surface area (b) in the synovial membrane. The data has been presented as a mean value ± standard deviation (SD). ^∗^*p* < 0.05. OA: osteoarthritis; HA: hemophilic arthropathy.

The evaluation of the number and surface area of blood vessels was performed with specific antibodies against vascular endothelium (CD31^+^). Welch *t*-test was conducted in order to compare these parameters of two investigated groups. Therefore, the immunolabeling was performed only in the synovial membrane due to the lack of blood vessel presence within the articular cartilage (Figure 6). The analysis showed significant (*p* = 0.0279) differences in the average number of blood vessels between groups with the predominance of preparations sampled from the control group patients (Figure 7). The average number of blood vessels was estimated at 65.7 ± 20.16 vessels per HPF in the synovial membrane obtained from study group patients, while in the control group, the respective value was estimated at 78.8 ± 15.71 vessels per HPF. Consecutively, the surface area of blood vessels was larger in study group patients with the average of 0.54 ± 0.050 fraction of the total surface area while the respective value in control group patients was 0.52 ± 0.039 fraction of the total surface area. The average values concerning the surface area of blood vessels showed no statistically significant (*p* = 0.6666) differences.

#### 3.2.4. Comparative Assessment of the Articular Cartilage Degeneration and Fibrosis

The obtained results were classified according to the present authors' own semiquantitative scale. The Fisher test was conducted in order to compare these parameters of two investigated groups. No statistically significant (*p* = 0.571) differences were noted as regards the articular cartilage fibrosis in the study and control groups. The observed lesions exceeded ~50% in both cases. The degree of articular cartilage degeneration reached almost ~100% of the surface area in some cases in both groups, with more exacerbation observed in patients from the study group. However, the results were not statistically significant (*p* = 0.154), most probably due to the low number of samples.

#### 3.2.5. Comparative Assessment of the Synovial Membrane Proliferation and Fibrosis

The analogous statistical analysis was conducted to determine the synovial membrane proliferation and fibrosis grade. In the case of the synovial membrane of control group patients, the degree of proliferation did not exceed ~25% (grade 1). Therefore, in the case of the study group patients, over ~75% of the analyzed cases showed the signs of cell proliferation estimated at the level of 50-75% (grades 2 and 3). The average values concerning synovial membrane proliferation showed statistically significant (*p* = 0.000000377) differences in this case. The synovial membrane fibrosis occurred both in the study and control group patients, with the process being more exacerbated in the study group patients, and in ~75% of analyzed cases, it exceeded 50% (grade 3) which constituted a statistically significant (*p* = 0.0307) difference.

## 4. Discussion

The pathophysiology of HA establishes a hallmark of heterogeneous mechanisms related with the joint degeneration which has not been fully elucidated especially in reference to the development of inflammatory and catabolic processes occurring within the articular cartilage and synovial membrane. In recent years, the wide range of the mediators was attributed with HA pathogenesis including cytokines along with the chemokines and their associated receptors and signaling pathways. In the following study, we have confirmed the participation of the CX3CL1/CX3CR1 signaling axis in the development of degenerative joint lesions in HA patients. Consecutively, we have shown a statistically significant (*p* = 0.000078) elevated average concentration of CX3CL1 in the serum of the study group (7.16 ± 0.53 ng/ml) of HA patients compared to the control group (5.85 ± 0.70 ng/ml) of OA patients. Although we did not show any statistically significant (*p* = 0.251) difference in other examined biofluids such as synovial fluid concerning the concentration level of CX3CL1 between the study group (4.81 ± 1.39 ng/ml) of HA patients and the control group (4.38 ± 1.08 ng/ml) of OA patients, a higher mean CX3CL1 concentration level was reported in HA patients. Potentially, such observations could in this case also result from the insufficient and small number of the study and control group participants. The immunohistochemical and histological stainings showed the CX3CR1 overexpression in the synovial membrane which is comparable in both the study and control groups. No overexpression of CX3CR1 was noted in the slices of the articular cartilage between groups. However, an increased tendency towards the staining of the extracellular matrix was observable without significant differences between the study and control groups. The obtained data indirectly show that inflammatory and degenerative processes mediated by CX3CR1 activation demonstrate similar intensity in the synovial membrane of affected joints in both groups of patients. However, both the activity of synovial membrane cells concerning CX3CL1 production and the systemic tendency towards its production by immune system cells referred to as its serum concentration are distinctly more marked in HA patients. A similar level of CX3CR1 overexpression in the slices of the synovial membrane may be partly accounted for by the similar stage of clinical development of the joint disease in both groups and also due to the appropriate preoperative regular administration of FRT to HA patients and introduction of an appropriate care program which minimizes the risk of hemarthrosis and exacerbations of joint-related manifestations [24]. It may not be ruled out that this could contribute to the partial reduction in CX3CR1 expression in HA patients observed in the present study. Seemingly, the assessment of the expression of CX3CR1 would be the optimal solution in these patients immediately after the episode of acute hemarthrosis, but conducting such a study would be clearly limited. Therefore, considering the previous research on a preclinical rodent model of hemophilia A, it may be indirectly assumed that CX3CR1 expression could increase throughout as it was observed in the case of other inflammatory cytokines interconnected with the CX3CL1/CX3CR1 axis, such as interleukin 1 beta (IL-1*β*) and tumor necrosis factor alpha (TNF*α*) [25]. Notably, CX3CR1 overexpression occurs only in synovial membrane slices, while in articular cartilage, CX3CR1 expression is not increased. It may be consistent with an accepted view that degenerative lesions of the articular cartilage are triggered by active immune processes occurring in the synovial membrane [26]. The presented data seem to confirm that the synovial membrane in OA and also in HA patients constituted the main inflammatory focus leading to the progressive impairment of the function and destruction of affected joints. It correlates with our observations regarding the CX3CR1 overexpression in both groups of patients in the slices of the synovial membrane compared to articular cartilage. Furthermore, the obtained data concerning CX3CL1 concentration in the blood serum and synovial fluid in OA patients seem to correlate with the previously observed findings reported by other researchers [27–29]. Therefore, the results may be indirectly confirmed and assumed that the methodology of the present study was appropriate and reliable. Moreover, it confirms the validity of the present results of CX3CL1 concentrations in the blood serum and synovial fluid in HA patients, as such values had not been evaluated previously. The presence of numerous macrophages that formed the observed foci indicates on the exacerbated inflammatory process occurring in the synovial membrane in HA patients whereas its recruitment is mediated by the CX3CL1/CX3CR1 axis [30]. It needs to be emphasized that the total number of macrophages (CD68^+^) in the slices and per HPF was higher in the study group compared to the control group with the statistically significant difference in the total number of CD68^+^ cells. Macrophages consist of cells that express CX3CR1 and are sensitive to the chemotactic activity of the free and bound forms of CX3CL1. The assembly of CX3CL1 and CX3CR1 with assistance of other endothelial cells adhesive molecules allows immune cells to penetrate through blood vessels into the site of ongoing inflammation [31]. The increased number of CD68^+^ cells in the synovial membrane of HA patients is most probably associated with the increased average concentration of CX3CL1 in the blood serum and synovial fluid compared to OA patients. This may contribute to the marked intensification and acceleration of degenerative processes in the articular cartilage and periarticular tissue, which is reflected by the average age (37.4 vs. 48.6 years) of patients undergoing surgery in both groups. The cytotoxic activity occurring via direct activation of NK cells (CD56^+^) is one of the numerous properties mediated by the CX3CL1/CX3CR1 axis [32]. This specific subpopulation lymphocytes cause the rapid apoptosis of target cells resulting in the destruction of surrounding tissues via degranulation and consecutive secretion of perforins, granulysin, and granzymes [33]. Therefore, the obtained slices of the articular cartilage and synovial membrane were tested for the presence of this type of cells. No statistically significant differences were demonstrated, but it needs to be emphasized that the presence of solitary NK cells was reported only in the synovial membrane of HA patients (0.8 of the cell per HPF). More developed degenerative lesions of the synovial membrane were observed in HA patients than in OA patients. The synovial membrane of 75% HA patients showed the signs of excessive cell proliferation (grade 3), while in OA patients, it did not exceed 25% (grade 1). Similarly, in the case of the synovial membrane fibrosis in HA patients, the lesions were more exacerbated than in the control group patients. Notably, statistical significance was reported for the obtained results concerning the degree of proliferation and fibrosis of the synovial membrane. Both these processes result from the excessive activation and interplay of immune system cells and joint-forming cells [34]. Catabolic processes which damage tissues, mediated by factors such as the CX3CL1/CX3CR1 axis, in this case stimulate pathological repair predominantly involving the deposition of collagen fibers and scarring process which clinically present as joint edema resulting in limiting its mobility and substantial pain [35]. Apart from the synovial membrane proliferation and fibrosis, degenerative joint lesions are also manifested by the articular cartilage degeneration and fibrosis, both in patients with HA and OA. Consecutively, the present study has not shown significant differences as regards the fibrosis and defects of the articular cartilage between the study group and the control group. However, it needs to be emphasized that the lesions were more marked in the slices obtained from HA patients. The results appear not to be unexpected, taking into consideration the enrollment criteria for the study that included individuals requiring arthroplasty procedures, classified mainly as grade 4 due to the Kellgren-Lawrence scale in both the study and control groups. Despite the lack of statistically significant intergroup differences concerning the articular cartilage degeneration and fibrosis, it should not be assumed that the role of inflammatory cytokines, including the CX3CL1/CX3CR1 axis, is comparable in this case. The age of onset of such grade of exacerbation of articular lesions is the principal factor differentiating both the examined groups. We observed that the onset of HA occurred on average 11 years and almost 3 months earlier compared to the control group patients suffering from OA. It may indicate in this case the considerably higher hyperactivity of the CX3CL1/CX3CR1 axis, indirectly indicating the early onset of joint destruction requiring prior specialist surgery over the significantly shorter period of time. The last parameters that were evaluated in the present study covered the assessment of the possible role of the CX3CL1/CX3CR1 axis on the hyperplasia of the vascular endothelium (CD31^+^). It was demonstrated that CX3CR1 occurring in the endothelial cell surface is responsible for the activation of angiogenesis in two independent mechanisms via the stimulation of expression for genes of hypoxia-inducible factor-1 alpha (HIF-1*α*) and vascular endothelial growth factor (VEGF) [36]. Increased VEGF activity is observed in the numerous joint degenerative diseases, including HA and OA [37, 38]. In case of the excessive activation of inflammatory pathways, including the CX3CL1/CX3CR1 axis, the pathological hyperplasia and angiogenesis may occur which result in the increased tissue perfusion and promotion of phenomena connected with the penetration of immune system cells stimulating further catabolic processes [39]. The described phenomenon plays the significant role, especially in the synovial membrane of affected joints [40]. The obtained results indicate the presence of statistically significant differences in the average number of blood vessels between groups. The higher average number of blood vessels was noted in OA patients (78.8 ± 15.71 vessels per HPF) compared to in HA patients (65.7 ± 20.16 vessels per HPF). Conversely, the larger average surface area of blood vessels was noted in HA patients (0.54 ± 0.039) compared to OA patients (0.52 ± 0.050). However, most probably due to the small size of the group, statistically significant differences for these results were not noted. The presence of angiogenesis in the joint degenerative diseases is mainly associated with the concomitant secondary regenerative processes [41, 42]. The higher number of blood vessels was observed in the synovial membrane of OA patients which indirectly results from the nature of the disease. Alternating the occurrence of exacerbations and remissions is characteristic of OA compared to HA which has a rather chronic progressive course and much less characteristic remission periods [43, 44]. As regards HA patients, the metabolism of eliminating erythrocyte-derived iron (Fe^2+^) and deposits of hemosiderin is a long-lasting process, which contributes to the absence of remission [45]. Therefore, with the time of the progression of disease, the inflammatory and degenerative processes gradually overlap regenerative processes, including the angiogenesis which may result in the lower intensity of developing new blood vessels [8, 46]. The synovial membrane in HA patients is markedly fibrous and rigid as a result of a long-lasting chronic inflammatory process mediated potentially by such factors as the CX3CL1/CX3CR1 axis. This may mechanically impede the process of development and growth of new blood vessels. In this case, the activity of growth factors stimulating angiogenesis may trigger the hyperplasia of vessels which already exist, what is reflected as an observed increased surface area of synovial membrane vessels in HA patients. The structure of the synovial membrane is markedly less degenerated and rigid in OA patients, so the angiogenesis may be much more commonly manifested by the development of new smaller blood vessels. The present results indicate the participation of the CX3CL1/CX3CR1 axis in the pathogenesis of HA. Furthermore, the action of this signaling axis is present in both groups with the seemingly more marked influence observed in patients with HA. It is particularly visible in the statistically significant results such as an increased concentration of CX3CL1 in blood serum, an increased macrophage count in tissue slices, and a more marked proliferation and fibrosis of the synovial membrane in HA patients. The increased CX3CL1 concentration in the synovial fluid, presence of NK cells, and larger surface area of blood vessels within the synovial membrane were noted in the same group of patients. However, the observed differences were not statistically significant compared to those of the control group. The main reason why the appropriate significance level was not reached was the relatively small size of the study group caused by less and less common advanced fully developed form of HA in patients with a severe form of hemophilia A due to the use of early regular administration of FRT [47]. It needs to be emphasized that obtaining tissue samples from the study group (especially intraoperative material) is very complicated in Polish conditions because of the few highly specialized centers which offer comprehensive treatment for HA patients. Orthopedists still consider such surgeries as associated with the increased risk of excessive perioperative bleeding and other serious complications, even if appropriate prophylaxis is provided. The arthroplasty procedures are performed very rarely and with particular safety measures in such patients. Therefore, despite the small size of the study group, the obtained sample material seems to be valuable from the scientific viewpoint. HA patients do not constitute the largest group of individuals suffering from degenerative joint diseases, but the course of the disease should be viewed as the most severe [48]. The patients experience mobility impairment, which is frequently much more severe than in OA patients. The majority of the patients aged over ~30 years require orthopedic surgeries to avoid disability. All the research focusing on the improvement of the health-related quality of life (HRQoL) in this group of patients, especially concerning the methods of slowing down the progress of the disease and reducing pain, should be considered as highly useful. The present study points out the potential influence and involvement of the CX3CL1/CX3CR1 axis in the exacerbation of degenerative lesions in the joints of HA and OA patients. It concentrates on the most possible pathomechanisms of these processes in the context of currently available knowledge. We think that the complete elucidation of the role of CX3CL1 and CX3CR1 in the pathogenesis of HA and other joint pathologies necessitates much more research based on reliable methodology and larger patient groups and studies including experimental models to demonstrate the specific role of the CXRCL1/CX3CR1 axis in hemophilic arthropathy. It is the only way for in-depth elucidation of the mechanisms underlying this type of pathologies and also the key to the development of effective and safe prophylaxis and treatment methods [49]. Collectively, the observations using selected methods and stainings support the point of view that evaluation of CX3CL1/CX3CR1-related parameters possesses the potential improvement and the new interesting tool for the future investigations and research in joint degenerative disorder diagnosis.

## 5. Conclusions

This study has demonstrated collective CX3CL1/CX3CR1 axis involvement in hemophilic arthropathy pathogenesis. This study also provides additional information about occurrence and the possible role of CX3CL1/CX3CR1 axis-related cells in joint tissue degeneration such as macrophages, fibroblasts, NK cells, or endothelial cells. An increased concentration of the CX3CL1 chemokine in blood serum, an increased macrophage count in histological material, and a higher level of fibrosis with proliferation of the synovial membrane were observed in HA patients comparing to OA patients (*p* < 0.05). Additionally, HA patients were characterized by increased CX3CL1 chemokine concentration in the synovial fluid, the presence of NK cells, and a larger surface area of blood vessels in the synovial membrane, but the observed differences were not statistically significant compared to those of the control group (*p* > 0.05). Based on the literature review, there is no similar study that has been previously published. Complete elucidation of the role of CX3CL1 and CX3CR1 in the pathogenesis of HA and other joint pathologies necessitates more research and may be promising targets to develop effective and safe treatment methods of these kinds of pathologies.

## Figures and Tables

**Table 1:** The semiquantitative scale implemented for the assessment of the articular cartilage and synovial membrane degree of lesions in analyzed tissue slices per HPF.

Grade	Percentage of the occupied changed tissue slice surface
0	0
1	1-25%
2	26-50%
3	51-75%
4	76-100%

## Data Availability

The datasets used and/or analyzed in this study are available from the corresponding author on reasonable request.
