# Corrigendum to “Comparative Analysis of the Occurrence and Role of CX3CL1 (Fractalkine) and Its Receptor CX3CR1 in Hemophilic Arthropathy and Osteoarthritis”

**DOI:** 10.1155/2020/7179283

**Published:** 2020-11-14

**Authors:** Piotr Wojdasiewicz, Łukasz A. Poniatowski, Andrzej Kotela, Marta Skoda, Michał Pyzlak, Aleksandra Stangret, Ireneusz Kotela, Dariusz Szukiewicz

**Affiliations:** ^1^Department of General and Experimental Pathology, Centre for Preclinical Research and Technology (CePT), Medical University of Warsaw, Pawińskiego 3C, 02-106 Warsaw, Poland; ^2^Department of Rehabilitation, Eleonora Reicher National Institute of Geriatrics, Rheumatology and Rehabilitation, Spartańska 1, 02-637 Warsaw, Poland; ^3^Department of Experimental and Clinical Pharmacology, Centre for Preclinical Research and Technology (CePT), Medical University of Warsaw, Banacha 1B, 02-097 Warsaw, Poland; ^4^Department of Neurosurgery, Maria Skłodowska-Curie Memorial Cancer Center and Institute of Oncology, W. K. Roentgena 5, 02-781 Warsaw, Poland; ^5^Faculty of Medicine, Collegium Medicum, Cardinal Stefan Wyszynski University in Warsaw, Poland; ^6^Department of Interventional Medicine with the Laboratory of Medical Genetics, Institute of Medical Sciences, Collegium Medicum, Jan Kochanowski University, Kielce, Poland

In the article titled “Comparative Analysis of the Occurrence and Role of CX3CL1 (Fractalkine) and Its Receptor CX3CR1 in Hemophilic Arthropathy and Osteoarthritis” [1], author Andrzej Kotela was affiliated to “Department of Orthopedics and Traumatology, 1st Faculty of Medicine, Medical University of Warsaw, Lindleya 4, 02-005 Warsaw, Poland” and “Department of Orthopedics and Traumatology, Central Clinical Hospital of the Ministry of the Interior and Administration, Wołoska 137, 02-507 Warsaw, Poland” which are incorrect; author Ireneusz Kotela was affiliated to “Department of Orthopedics and Traumatology, Central Clinical Hospital of the Ministry of the Interior and Administration, Wołoska 137, 02-507 Warsaw, Poland” and “Department of Rehabilitation in Disease of the Locomotor System, Faculty of Medicine and Health Sciences, Jan Kochanowski University, Kielce, Poland” which are incorrect.

The correct affiliations for these authors are the following:

1. Andrzej Kotela: Faculty of Medicine, Collegium Medicum, Cardinal Stefan Wyszynski University in Warsaw, Poland
2. Ireneusz Kotela: Department of Interventional Medicine with the Laboratory of Medical Genetics, Institute of Medical Sciences, Collegium Medicum, Jan Kochanowski University, Kielce, Poland

The corrected list of affiliations is shown in the author information above.
